# Improvement of optical and structural properties of ZIF-8 by producing multifunctional Zn/Co bimetallic ZIFs for wastewater treatment from copper ions and dye

**DOI:** 10.1038/s41598-024-66276-7

**Published:** 2024-07-04

**Authors:** Pooneh Vatani, Maryam Aliannezhadi, Fatemeh Shariatmadar Tehrani

**Affiliations:** Faculty of Physics, Semnan University, PO Box: 35195-363, Semnan, Iran

**Keywords:** Light and matter interactions, Metal–organic frameworks (MOFs), Optical properties, Zeolitic imidazolate framework, Bimetallic ZIFs, Hybrid materials, Applied physics, Nanoparticles, Environmental sciences

## Abstract

In the paper, high specific surface area (SSA) mono and bimetallic zeolitic imidazolate frameworks (ZIFs) based on zinc and cobalt metals are successfully synthesized at room temperature using different ratios of Zn to Co salts as precursors and ammonium as a solvent to tailor the properties of the produced ZIF and optimize the efficiency of the particles in water treatment from dye and copper ions, simultaneously. The results declare that monometallic and bimetallic ZIF microparticles are formed using ammonium and the tuning of pore sizes and also increasing the SSA by inserting the Co ions in Zn-ZIF particles is accessible. It leads to a significant increase in the thermal stability of bimetallic Zn/Co-ZIF and the appearance of an absorption band in the visible region due to the existence of Co in the bimetallic structures. The bandgap energies of bimetallic ZIFs are close to that of the monometallic Co-ZIF-8, indicating controlling the bandgap by Co ZIF. Furthermore, the ZIFs samples are applied for water treatment from copper ions (10 and 184 ppm) and methylene blue (10 ppm) under visible irradiation and the optimized multifunctional bimetallic Zn/Co ZIF is introduced as an admirable candidate for water treatment even in acidic conditions.

## Introduction

The treatment of polluted water containing organic dyes or metal ions such as methylene blue and copper is crucial due to the potentially harmful effects on the environment and human health. These pollutants can be present in drinking water sources or discharged into the life medium, posing risks to aquatic life and human health. Various treatment methods, including physical, chemical, and biological approaches, are being introduced to effectively remove these pollutants from waters, ensuring their safety for consumption and reducing environmental impact. Today, the applications of materials such as metal–organic frameworks (MOFs) specifically zeolitic imidazolate frameworks (ZIF) based on zinc or cobalt show excellent potential in water treatment applications, etc.^1–4^. These frameworks have demonstrated high efficiency in removing dyes and metal ions like copper due to their porous structure and large surface area for adsorption.

ZIF-8, a type of MOFs composed of zinc atoms coordinated with imidazolate ligands, has several advantages including high thermal stability, large surface area, tunable porosity, and chemical versatility. These advantages lead to retaining its structure and properties at high temperatures, providing abundant active sites for chemical reactions, obtaining selective gas adsorption or molecular sieving, and easily functionalizing by incorporating different metal ions or ligands during synthesis. It also causes the expansion of its potential applications for various applications like catalysis, selective gas adsorption or molecular sieving, separation processes, and gas storage. Despite its high thermal stability and special properties, its electromagnetic wave absorptions in optical ranges (ultraviolet, visible, and near-infrared) are limited which restricts its usage in some applications like water treatment from organic dyes by photocatalytic process and cancer treatment by photodynamic therapy (PDT). Different studies have been done on the structure to improve their properties, especially for applications based on photon absorptions^5–10^.

Bimetallic ZIFs are a type of hybrid material that combines the properties of two MOFs to achieve unique structural and chemical properties. They have gained significant attention in recent years due to their enhanced catalytic activities, improved stability and durability, and the synergistic effects between the two metals used in the structure leading to improving the advantages of materials compared to individual metals or monometallic ZIFs. Tuning the properties of bimetallic ZIFs including tailoring the material's pore size, surface area, acidity/basicity, redox properties, and controlling their absorption is possible by selecting different metal combinations and ratios. Their tunable properties and enhanced performances make them valuable for addressing challenges in these areas.

Different ZIF-8 composites have been introduced as excellent materials for pollutant removal or photodegradation including dyes or antibiotics from wastewater and detailed information about them can be found in the references^11,12^. For example, Ag_3_PO_4_@ZIF-8 heterojunction has been provided by chemical precipitation and used for the photodegradation of organic pollutants including organic pollutants crystal violet, congo red, and rhodamine B. Their results displayed a wonderful photocatalytic activity of the synthesized material^13^. Furthermore, ZIF-8@GO-COOH composite has been prepared and applied for water treatment by adsorptions of Cu and Pb from water and their results demonstrated the high adsorption performance of the composite^14^. Co-doped Zn ZIF has been synthesized using agar powder. Then photocatalytic activities of the ZIF-8 and 10% Co-doped ZIF-8 by photodegradating methylene blue (MB) under visible light have been studied^15^. Their results indicated that the photocatalytic activities of the samples rise with dopping Co atoms in the structure. Also, Co-doped Zn ZIF nanoparticles with particle sizes in the range of 150–300 nm have been provided and the enhancement in CO_2_ and H_2_ uptakes was observed compared to Zn ZIF^16^. Also, synthesizing ZIF-67 and MCM-41/ZIF-67 composites by water solvent at room temperature showed that the thermal stability and adsorption capacity of MC (10)/ZIF-67 is more than ZIF-67^17^. Finally, it can be understood that tailoring and synthesis of bimetallic ZIFs are complex due to the dependence of their properties on various factors including the choice of metals, synthesis conditions, post-synthesis modifications, etc. However, the potential benefits of the bimetallic ZIFs make them an attractive research area with promising applications in the simultaneous treatment of water from several pollutants.

From the mentioned research, it can be concluded that the synthesis of bimetallic ZIFs with different metal combinations and ratios can lead to tuning the properties of bimetallic ZIFs including control of the optical responses, the material's pore size, surface area, etc. which is required for a wide range of applications in the environment, medicine, and industry specifically water treatment. Monometallic ZIFs based on zinc or cobalt offer several advantages for water treatment from dyes and metal ions, including high adsorption capacity, selectivity, renewability, stability, and scalability. Researchers continue to explore and optimize the use of ZIFs in water treatment to address emerging environmental challenges effectively. The ZIFs can be tailored to optimize their performance for specific pollutants, including dyes and metal ions like copper. Therefore, in this paper, the effects of synthesizing bimetallic ZIFs using ammonium hydroxide aqueous solution with different molar ratios of zinc salt to cobalt salt are investigated to tailor the ZIF structures and improve the adsorption efficiency and photocatalytic activities of the structure for water treatment from dye and metal ions pollutants. The results demonstrate the significant effects of the salt molar ratio on the properties of bimetallic ZIF particles and optimized ZIF is introduced as multifunctional particles for water treatment from dyes and metal ions.

## Materials and methods

### Synthesis

Zinc nitrate hexahydrate (Zn(NO_3_)_2_,6H_2_O), cobalt nitrate hexahydrate (Co(NO_3_)_2_·6H_2_O), 2-methylimidazole (Hmim, C_4_H_6_N_2_), ammonium hydroxide aqueous solution (NH_4_OH, 25% aqueous solution), methanol (CH_3_OH), methylene blue, and copper (II) nitrate (Cu(NO_3_)_2_·3H_2_O) were purchased from Merck company with the purity of 99.9% and used without any purification in the synthesis of single- and bi-metallic ZIF samples and their application in water treatment.

To synthesize the Zn-ZIF, first, 0.594 g of zinc nitrate hexahydrate (2 mmol) was dissolved in 3 mL of deionized (DI) water, and 2-methylimidazole (4 mmol) was dissolved in 4.8 mL of Aluminum hydroxide solution (NH_4_OH), separately. Second, two solutions were mixed and put on a magnetic stirrer for 10 min at room temperature to obtain a milky solution. The third step is related to washing the sample which was done by centrifuging the white solution and washing it twice with DI water and then once with methanol. Then, the product was kept in methanol for 1 day followed by centrifuging and drying in the oven at 60 °C for 12 h to obtain the white powder. The sample was labeled as the Zn-ZIF sample and different analyses were done on the sample.

For the synthesis of the ZIF sample based on cobalt, the same process with the same molarity was done using cobalt nitrate hexahydrate, and purple powder was labeled as Co-ZIF and analyzed. Also, different molar ratios percentages of zinc nitrate hexahydrate to cobalt nitrate hexahydrate (75:25, 50:50, and 25:75 percent) were established to synthesize bi-metallic composite ZIFs based on Zn and Co with the above conditions. It is worth mentioning that the total molarity of metal salts was fixed (2 mmol) in the synthesis process. However, the molar ratios of metal salts were changed in synthesized processes to obtain bi-metallic ZIF samples. The synthesized bimetallic ZIF samples were labeled according to molar ratio percents of Zn salt to Co salt as ZIF:75:25, ZIF:50:50, and ZIF:25:75. Finally, different analyses were done on them to find information about all synthesized samples.

### Characterization

TESCAN-model MIRA3 microscope and Shimadzu spectrometer-model 8400S were established to determine the morphology, chemical elements, and chemical bonds formed in the mono- and bi-metallic ZIF samples based on Zn and Co. Also, the X-ray diffractometer- ADVANCE-D8 model with Cu-kα radiation source (λ = 1.5406 Å) and diffuse reflectance spectrometer- Avaspec-2048-TEC model were used to investigate the structural and optical properties of the synthesized ZIF samples, respectively. Indeed, Micromeritics Gemini VII version 5.03 and Linseis TGA PT1000 were employed to determine the specific surface areas (SSAs), pore distributions, and thermal stabilities of the ZIF samples.

### Application

To investigate the copper removal abilities of the synthesized mono- and bi-metallic ZIF samples, 10 and 184 ppm copper ion solutions were provided by dissolving Copper (II) nitrate in DI water at room temperature. Then, given values of ZIF samples (0.001 g for 10 ppm Cu^2+^ solution and 0.001, 0.0025, and 0.005 g for 184 ppm Cu^2+^ solution) were added to 50 mL of the given molarity of Cu^2+^ solution located on the magnetic stirrer at room temperature to provide the contact between adsorbate and adsorbent. After that, at the given contact times, 3 mL of the solution was centrifuged to separate the ZIF samples, and the solution was investigated using atomic absorption spectroscopy (AAS) to determine the concentration of copper ions in the solution and estimate the efficiencies of the mono- and bi-metallic ZIF samples for water treatment from copper ions as a very dangerous ion. Agilest Technologies 240AA flame atomic absorption spectrometers were established to determine the concentration of copper ions in the solutions during the water treatment at every given time and calculate the copper ion removal efficiency of the particles.

To estimate the ability of mono- and bi-metallic ZIF samples for water treatment from dye, the MB was selected, and 0.005 g of ZIF samples were added to the MB solution (10 ppm) located on the magnetic stirrer at room temperature and kept them in the dark condition for 20 min. After that, the white lamp irradiation (three 15 W incandescent lamps with a distance of 10 cm) was applied to the MB solution containing the ZIF sample and then 3 mL of the solution was separated at the given times, centrifuged to separate the ZIF particles. Then, a homemade visible spectrometer determined the absorbance of the solution leading to determining the concentration of MB in the solution and calculating the efficiency of the mono- and bi-metallic ZIF in water treatment from dye (MB) under visible irradiation.

## Results and discussion

In this section, the results related to the characteristics of mono- and bi-metallic ZIF samples based on zinc and cobalt are presented to obtain some information about the samples and also study the effects of providing Zn/Co bimetallic ZIF with different molar ratios. Then, the photocatalytic activities of mono- and bi-metallic ZIF particles are presented using photodegradation of MB dye by the ZIFs under visible light. In the following, the abilities of mono- and bi-metallic ZIF particles to treat the water from copper ions as a dangerous metal are also evaluated.

### Morphology

**Figure 1:**
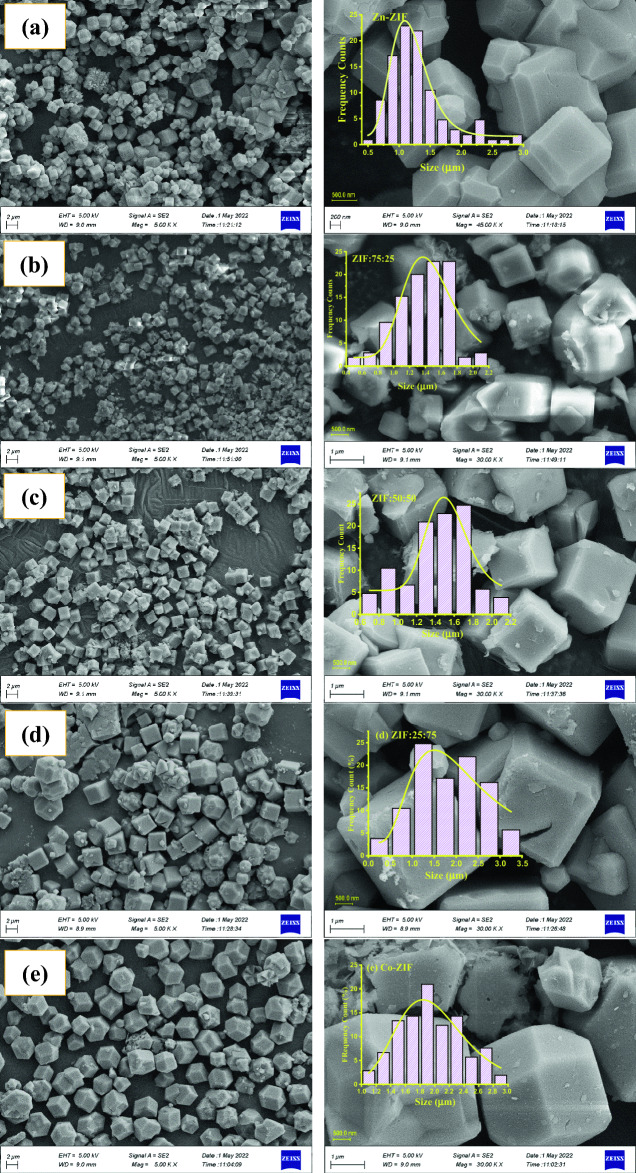
Images of mono and bi-metallic ZIF samples with zinc or/and cobalt with two different magnifications labeled as (**a**) Zn-ZIF, (**b**) ZIF:75:25, (**c**) ZIF:50:50, (**d**) ZIF:25:75, and (**e**) Co-ZIF taken by field emission scanning electron microscopy (FE-SEM). Histograms of size distributions and fitted log-normal functions to the histograms are depicted in the inset of the FE-SEM images of ZIF samples.

In Fig. 1, field emission scanning electron microscopy (FESEM) images of mono- and bi-metallic ZIF samples based on zinc or/and cobalt with different molar ratios of Zn to Co are shown at two different magnifications to consider the uniformity and morphology of the ZIF samples. As you can observe, both monometallic bimetallic ZIF samples (containing Zn and Co) are multifaceted structures with a relatively uniform overall distribution. Also, according to Fig. 1a, cubic nanoparticles with truncated-faceted and truncated edges were formed in the Zn-ZIF. However, the morphology of the Co-ZIF sample in Fig. 1e is dodecahedrons (12 facets) with rhombus-shaped facets. Also, Fig. 1b–d demonstrate that the morphologies of formed bimetallic ZIF with Zn and Co are cubic particles with truncated edges. However, the particle sizes of bimetallic ZIF particles change with the change in the molar ratio of zinc to cobalt precursor used in the synthesis process. Furthermore, the most uniform size of bimetallic ZIF particles is observed for ZIF:50:50 with the same ratio of zinc to cobalt precursors (50:50). A qualitative comparison of the Zn-ZIF and Co-ZIF:0 shows that the monometallic Zn-ZIF particles have smaller dimensions compare to the Co-ZIF particles. The morphology of the formed particles can be connected to the Miller planes associated with the crystals of the samples. Formation of the cubic particles can be connected to [100] planes and rhombic dodecahedra particles can be connected to [100], [110], [111], and [211] planes^18,19^ which should be investigated by analyzing the XRD patterns of the samples.

To consider the average sizes of the multifaceted structures formed in mono- and bi-metallic ZIF samples containing zinc or/and cobalt, the Digimizer software was used to measure the sizes of particles in every sample and the following log–normal function (Eq. 1)^20,21^ was fitted to calculate the average sizes (Eq. 2) and data dispersions (Eq. 3).

1 $$y(x) = y_{0} + \frac{A}{{\sqrt {2\pi } wx}}\exp \left( {\frac{{\ln (\frac{x}{{x_{0} }})^{2} }}{{2w^{2} }}} \right)$$

2 $$\overline{L} = x_{0} \exp \left( {\frac{{w^{2} }}{2}} \right).$$

3 $$\sigma_{L} = \overline{L} (e^{{w^{2} }} - 1)^{1/2}$$

where *x* and *y*(*x*) are the sizes of facets and their frequency counts in the ZIF samples, *y*_*o*_, *x*_*0*_, *A*, *x*, and *w* are fitting parameters of a log–normal function, and $\overline{L}$ and $\sigma_{L}$ are the obtained average facet length and data dispersion of the particle sizes for mono- and bi-metallic ZIF based on Zn and Co. The obtained histograms of facet size distributions for mono- and bi-metallic ZIF samples with zinc or/and cobalt and also the fitting log–normal function (solid yellow lines) are depicted in the inset of the FESEM image of every sample in Fig. 1a–e, and the average sizes, data dispersions, and percentage of data dispersion are collected in Table 1. As can be observed, the average size of the facet in Zn-ZIF increases by increasing the nominal value of Co in the structures and decreasing the average value of the facet size from ZIF:25:75 to Co-ZIF is related to the change in the morphology of the particles from truncated-edge cubic to dodecahedrons with rhombus-shaped facets. Furthermore, the most uniform multifaceted hollow particles are formed using the same percentage of Zn and Co salts in the ZIF:50:50 sample.

**Table 1:** The average facet sizes, data dispersions, and data dispersion percentages of multifaceted hollow structures formed in mono- and bi-metallic ZIF samples containing zinc and cobalt.

Sample	Average size ($\overline{L}$) (µm)	Data dispersion (*σ*_*L*_) (µm)	Percentage of data dispersion (%)
ZIF-8	1.200	0.039	3.3
ZIF:75:25	1.473	0.037	2.5
ZIF:50:50	1.534	0.0158	1.0
ZIF:25:75	2.259	0.353	15.6
ZIF-67	2.004	0.059	2.7

### Chemical elements

**Figure 2:**
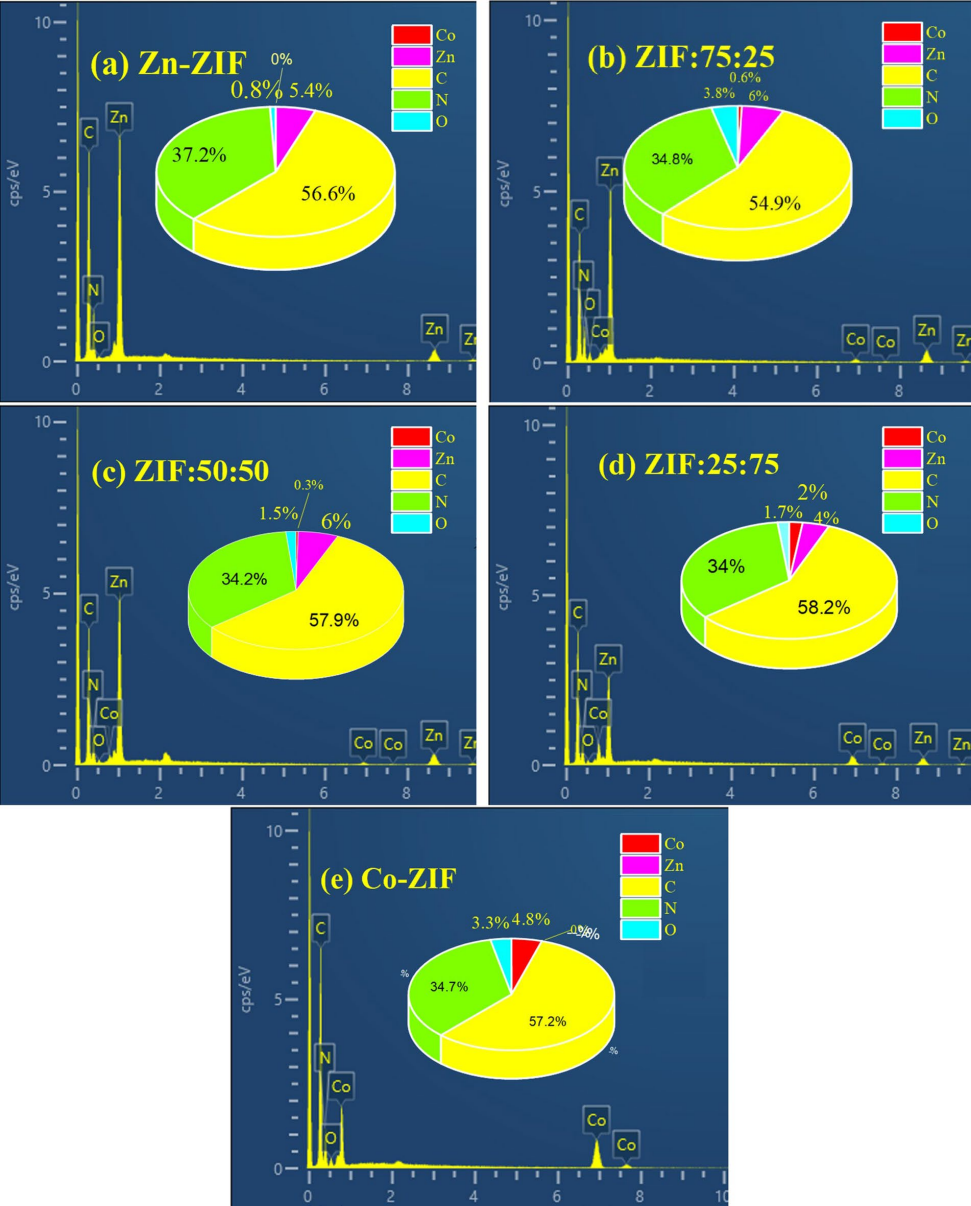
EDX spectra of mono- and bi-metallic ZIF samples with zinc or/and cobalt with the different molar ratios of Zn to Co labeled as (**a**) Zn-ZIF, (**b**) ZIF:75:25, (**c**) ZIF:50:50, (**d**) ZIF:25:75, and (**e**) Co-ZIF taken by field emission scanning electron microscopy. The atomic percentages of chemical elements are displayed in the inset of figures for every mono- and bi-metallic ZIF sample.

EDX spectra of mono-and bi-metallic ZIF samples based on zinc or/and cobalt with the different molar ratios of Zn to Co salts are depicted in Fig. 2a–e and the atomic percentages of chemical elements are also plotted in the inset of every figure. As it can be observed, no impurity can be observed in the mono- and bi-metallic ZIF synthesized using ammonium and all synthesized samples are pure. Although, the atomic percentages of Zn and O in the mono-metallic ZIF particles and Zn: Co bimetallic ZIF samples change with a change in molar ratio, atomic percentages of C, O, and N in the samples are partly the same. Furthermore, atomic percentages of Zn in all ZIF composites, even in the ZIF:25:75 sample, are higher than this value for Co element which means that zinc has a greater tendency than cobalt to react and thus enter more in the structure of bimetal ZIF particles.

### Chemical bonds

**Figure 3:**
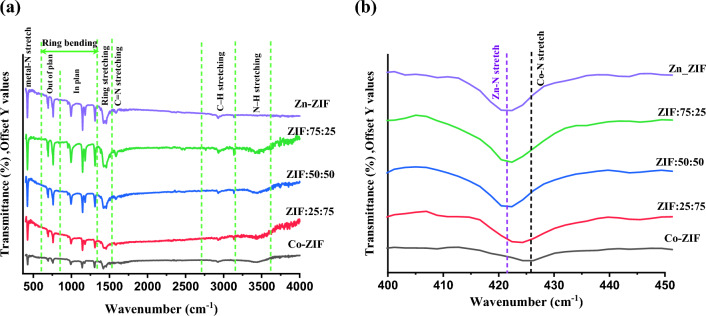
FTIR spectra of mono- and bi-metallic ZIF samples with zinc or/and cobalt with the different molar ratios of Zn to Co in (**a**) the range of 400–4000 cm^−1^, and (**b**) the fingerprint region of metal-nitrogen bands.

The mono- and bi-metallic ZIF samples based on Zn or/and Co were investigated by FTIR spectrometer to find some information about the organized chemical bonds in the samples and the FTIR spectra of the synthesized ZIF samples are plotted in Fig. 3a in the range of 400–4000 cm^−1^. The FTIR absorption peaks at 422 cm^−1^ and 426 cm^−1^ in the Zn-ZIF and Co-ZIF samples are attributed to Zn–N and Co–N stretching modes which are illustrated more clearly in Fig. 3b. The absorption peak experiences a blue shift in bimetallic ZIF samples due to increasing the Co in the Zn-ZIF structure.

Although mono- and bi-metallic ZIF particles have complex natures leading to difficulty in interpreting their FTIR absorption peaks, most of these absorption bands are related to vibrations of the imidazole and can be labeled based on them. The FTIR absorption bands in the range of 630–800 cm^−1^ and 950–1350 cm^−1^ are due to out-of-plane and in-plane bending of the rings^22^. Also, the absorption peak that appeared at ~ 1585 cm^−1^ of mono- and bi-metallic ZIF particles can demonstrate the existence of C=N stretching mode^22^. Indeed, the absorption peaks in the ranges of 1350–1500 cm^−1^ and 2890–3199 cm^−1^ can be denoted by the entire ring stretching and the C–H (aromatic and aliphatic) stretching modes^23^. Furthermore, the FTIR absorption peak in the range of 3200–3600 cm^−1^ can be attributed to the N–H stretching mode.

Therefore, it can be concluded from the FTIR spectra of the samples that mono- and bi-metallic ZIFs based on Zn or/and Co are successfully produced through the synthesis process.

### Crystallinity

**Figure 4:**
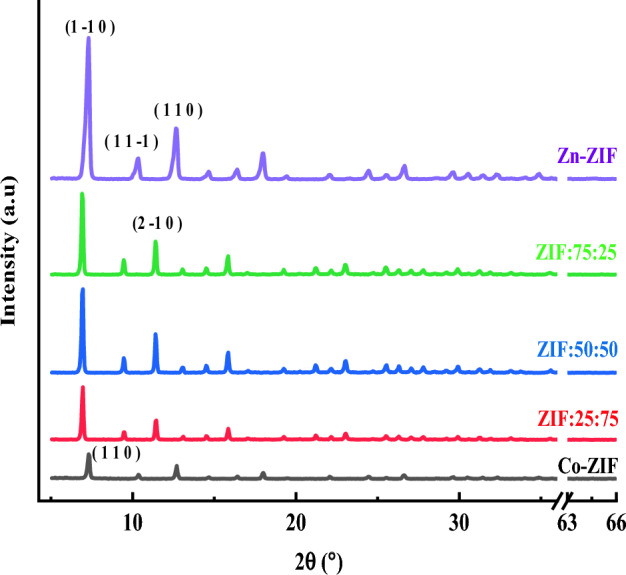
XRD patterns of mono-metallic and bi-metallic ZIF samples with zinc or/and cobalt ions with the different molar ratios of Zn to Co salts.

The mono- and bi-metallic ZIF samples with Zn or/and Co elements were examined using X-ray diffraction (XRD) in the range of *2θ* = 5°–65° to find some information about the crystalline features of the ZIF particles and the results are shown in Fig. 4. The existence of highly sharp and intense XRD peaks in the mono- and bi-metallic ZIF samples indicates a high percentage of crystallinity and the formation of large-size crystallite in the prepared ZIF samples. the dominant peak in Zn-ZIF is the peak that represents the (100) plane and shows that ZIF-8 tends to this orientation. On the other hand, the dominant peaks in Co-ZIF particles are related to (1–10) and (110) planes. The dominant peaks of bi-metallic particles are a combination of these two phases. Also, the XRD patterns show a gradual decrease in the intensity of the XRD peaks of the Zn-ZIF sample by inserting Co in the structure and providing Zn/Co bimetallic ZIF. However, the full width at half maximum (FWHM) of the main peak of the XRD pattern should be precisely considered to obtain some information about the change in the crystallinity of the Zn-ZIF sample by inserting Co in the structure and providing Zn/Co bimetallic ZIF samples.

The crystal phase of the synthesized mono- and bi-metallic ZIF particles based on Zn or/and Co were identified using the X’Pert high score software, and the results along with the matched standard cards, and chemical formulas are represented in Table 2. As can be observed, Zn-ZIF and Co-ZIF samples were formed in the anorthic and cubic phases, respectively, and the formations of crystallite bimetallic ZIF structures with Zn and Co metals in these mentioned phases are demonstrated. Therefore, bi-metallic Zn/Co ZIF samples have the same phases, and providing the composites of Zn/Co ZIFs with different ratios of Zn to Co salt causes no change in the phase of the synthesized samples. To consider the effects of inserting Co on the crystallite sizes of the Zn-ZIF samples, the Scherrer (*d*_*Sh*_ in Eq. 4) and Williamson–Hall (W–H) (*d*_*W–H*_ in Eq. 5) methods as two well-known methods for calculating the average crystallite size of the samples are used^24,25^.

4 $$d_{Sh} = \frac{k\lambda }{{\beta_{0} \cos \theta_{0} }}$$

5 $$\beta \cos \theta = \frac{K\lambda }{{d_{W - H} }} + 4\varepsilon \sin \theta$$

where *K* and *λ* appeared in Eqs. (4) and (5) are the shape factor (~ 0.94) and the irradiated X-ray wavelength, respectively. *ɛ* is also denoted as the micro strain of the structures. *θ* and *θ*_*0*_ are the Bragg angles of every peak in the W–H method and the main diffraction peak of the Scherrer method, respectively. Also, *β* and *β*_*0*_ are the FWHM of every peak in the W–H method and the FWHM of the main peak for the Scherrer method, respectively.

**Table 2:** Crystalline properties of multifaceted structures formed in samples with zinc or/and cobalt ions with the different molar ratios of Zn to Co salts.

Sample ID	Card number	Phase	Chemical formula	W–H crystallite size (nm)	Scherrer crystallite size for the main paek (nm)
Zn-ZIF	96-720-1499	Anorthic	C_36_H_36_N_24_Zn_6_	30.4	29.4
ZIF:75:25	96-720-1499 96-412-4529	Anorthic Cubic	C_36_H_36_N_24_Zn_6_ Co_12_N_48_C_96_H_120_	54.0	50.7
ZIF:50:50	96-720-1499 96-412-4529	Anorthic Cubic	C_36_H_36_N_24_Zn_6_ Co_12_N_48_C_96_H_120_	71.1	53.1
ZIF:25:75	96-720-1499 96-412-4529	Anorthic Cubic	C_36_H_36_N_24_Zn_6_ Co_12_N_48_C_96_H_120_	64.4	53.0
Co-ZIF	96-412-4529	Cubic	Co_12_N_48_C_96_H_120_	54.5	48.0

The average W–H crystallite size, *d*_*W–H*_, and micro stain, ε, of single- and bi-metallic Zn/Co ZIF samples can be calculated by fitting the linear function to $\beta \cos \theta$ versus $4\varepsilon \sin \theta$ data and determining the intercept and slope of the linear fit. The resulting average crystallite sizes of the samples are illustrated in Table 2. The results declare that the calculated average crystallite sizes by these two methods are almost matched together, and these average sizes show the same trend with inserting Co in the structure of Zn-ZIF particles and providing Zn/Co bi-metallic ZIF particles.

### Optical properties

**Figure 5:**
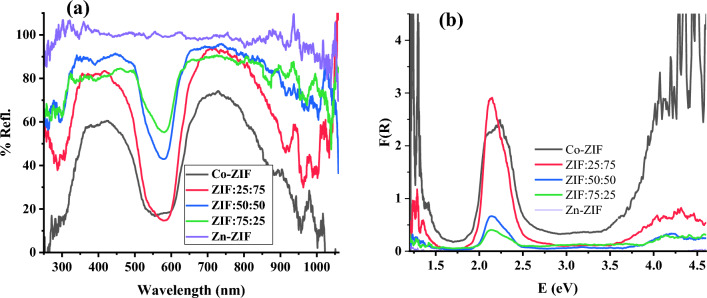
(**a**) DRS spectra, and (**b**) Kubelka–Munk function of samples with zinc or/and cobalt ions with the different molar ratios of Zn to Co salts.

Diffuse reflectance spectroscopy (DRS) was applied on the mono- and bi-metallic Zn/Co ZIF particles to study the optical properties of the ZIF samples and the effects of adding Co in the structures of Zn-ZIF particles and the reflectance are presented in Fig. 5a. The results declare that the reflectance of the Zn-ZIF particles (purple solid line) is almost 100% in the wavelength range of 300–1000 nm leading to proposing this sample for application required high reflection (HR) in the wide range from UV to NIR regions. Also, the results declare that the reflectance spectrum of Zn-ZIF particles experiences a significant change by adding the Co ions in the structure of these particles (providing Zn/Co bimetallic ZIF particles) such that the reflectance drops significantly in the wavelength region of 45–700 nm. This behavior becomes more intense for the samples containing larger amounts of Co ions. Also, the Kubelka–Munk function of ZIFs samples, $F(R) = (1 - R)^{2} /(2R)$^26,27^, which is proportional to the absorption coefficient of ZIF samples, is depicted in Fig. 5b which shows the presence of an absorption band in the 450–650 nm appeared due to inserting Co ions in the Zn-ZIF particles and providing Zn/Co bimetallic ZIF particles. Furthermore, the absorption edge of the Zn/Co bimetallic ZIF experiences a red shift with increasing the molar ratios of Co to Zn in the bimetallic ZIFs.

**Figure 6:**
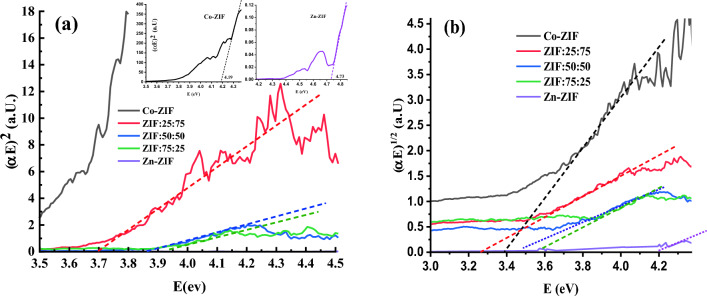
The Tauc plots of mono-and bi-metallic ZIF samples with zinc or/and cobalt ions with the different molar ratios of Zn to Co salts to calculate (**a**) direct, and (**b**) indirect bandgap energies.

To obtain the direct and indirect bandgap energies of mono-and bi-metallic ZIF samples with zinc or/and cobalt ions with the different molar ratios of Zn and Co, the following equation can be used to obtain Tauc plots which are depicted in Fig. 6, according to references^28^. The linear lines are fitted to the linear part of the Tauc curves to obtain the intersection of the line with the x-axis, and determine the band gap energy, *E*_*g*_, of every synthesized mono-and bi-metallic ZIF sample^29^:

6 $$(F(R)h\nu )^{2or0.5} = B(E - E_{g} )$$

where the power of 2 and 0.5 can be used to calculate the direct and indirect bandgap energies of the ZIF particles, respectively. Also, *B* is the constant and *E* is the applied photon energy.

The obtained direct and indirect bandgap energies of the mono- and bi-metallic ZIFs are gathered in Table 3, which indicates the decrease in bandgap energy of the Zn-ZIF sample by inserting the Co ions in the structure and providing the bi-metallic ZIF composites. Also, the bandgap energies of bimetallic particles are almost close to that of Co-ZIF which means the cobalt ions mainly control the optical properties of the bimetallic Zn/Co ZIF particles. Although the bandgap energy of Co-ZIF is partly near the reported value in^30^ for ZIF-67, the bandgap energies of Zn-ZIF and ZIF:50:50 are significantly lower than the reported values. This difference can be attributed to variations in synthesis procedures, solvent, and synthesis conditions, which have impacted the crystallinity of the formed ZIFs. There is no reported value for other investigated ZIF particles to compare with our current results.

**Table 3:** The direct and indirect bandgap energies of mono- and bi-metallic ZIF particles with zinc or/and cobalt ions with the different molar ratios of Zn to Co, calculated using the Tauc method.

Sample	Bandgap energies, E_g_(eV)	
	Direct	Indirect
Zn-ZIF	4.73	4.19
ZIF:75:25	3.92	3.46
ZIF:50:50	3.86	3.52
ZIF:25:75	3.74	3.33
Co-ZIF	4.19	3.4

### Pore sizes and specific surface area (SSA)

**Figure 7:**
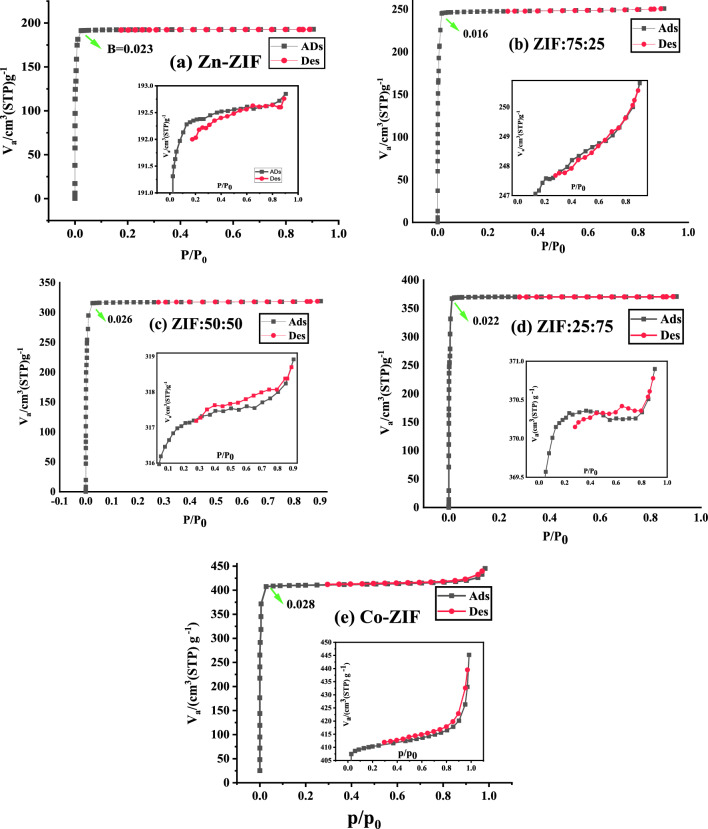
The nitrogen adsorption/desorption isotherms of mono- and bi-metallic ZIF samples with zinc or/and cobalt ions, and the different molar ratios of Zn to Co salts labeled as (**a**) Zn-ZIF, (**b**) ZIF:75:25, (**c**) ZIF:50:50, (**d**) ZIF:25:75, and (**e**) Co-ZIF. The hysteresis loops of the samples are enlarged for clarity and shown in the insets of the figures.

The nitrogen adsorption/desorption isotherms of mono- and bi-metallic ZIF samples with zinc or/and cobalt ions with different molar ratios of Zn or/and Co salts are presented in Fig. 7. Also, the hysteresis loops of the samples are enlarged for clarity and shown in the insets of Fig. 7. A sharp knee with a straight line can be observed in the nitrogen adsorption/desorption plots of all the samples, which indicates the filling of the first layer at low pressures (knee or B), as shown in Fig. 7a–e. These knees are related to type I of the IUPAC classification and suggest forming the micro pores in the mono- and bi-metallic ZIF samples. However, the Co-ZIF sample in Fig. 7e has a small hysteresis loop, which can also be regarded as type 2 of the IUPAC classification, meaning this sample is a mixture of type 1 and 2. The shape of the hysteresis loops of mono- and bi-metallic ZIF samples are consistent with the H3 and H4 types with a tendency towards one of them. ZIF:75:25 and Co-ZIF in Fig. 7b,e have a tendency toward the H3 type which means the dominant pore shape in these samples is slit-like pores. Other synthesized ZIF samples have a tendency towards the H4 type which means narrow slit-like pores and hollow spheres with walls composed of mesoporous are the dominant shape of the pores that can be related to filling the micropores or mesopores in the samples^31^.

**Figure 8:**
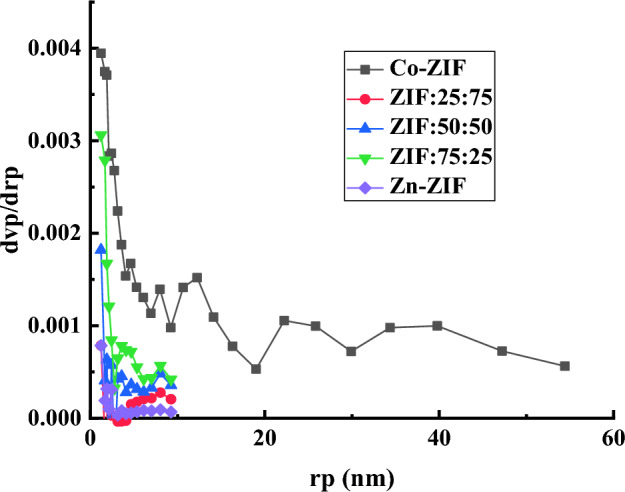
BJH pore size distribution plots of mono- and bi-metallic ZIF particles with zinc or/and cobalt ions, and the different molar ratios of Zn to Co salts derived from the desorption branch of isotherms.

The BJH pore size distributions of mono- and bimetallic ZIF particles with zinc or/and cobalt ions, and different molar ratios of Zn to Co are plotted in Fig. 8 indicating the formation of micropores structures in all ZIF samples which is consistent with the results obtained from nitrogen adsorption/desorption hysteresis loops of ZIF samples in Fig. 7 and their IUPAC classification. Also, inserting Co in the Zn-ZIF sample does not affect the ranges of the pore size distributions of the samples. The pore sizes of Zn-ZIF and bi-metallic Zn/Co ZIF samples are limited (less than 10 nm), while the pore size distribution of Co-ZIF particles is in the wide range up to 55 nm. However, the total pore volume of mono- and bi-metallic ZIF particles reported in Table 4 declares that inserting the Co ions in the Zn-ZIF structure leads to an increase in the total pore volume of the particles and a direct dependence between these quantities with increasing the cobalt ions in the samples can be observed. Therefore, the tuning of pore sizes by inserting the Co ions in Zn-ZIF particles is accessible.

**Table 4:** The SSA, mean pore diameter, and average pore volume of multifaceted structures formed in mono- and bi-metallic ZIF samples containing zinc or/and cobalt ions.

Sample	S_BET_ (m^2^/g)	Total pore volume (m^3^/g) (p/p_0_ = 0.904)	Mean pore diameter (nm)
Zn-ZIF	872.89	0.2983	1.3669
ZIF:75:25	1225.7	0.3879	1.2661
ZIF:50:50	1417.5	0.4933	1.392
ZIF:25:75	1567.0	0.5737	1.4644
Co-ZIF	1787.9	0.6887	1.5407

**Figure 9:**
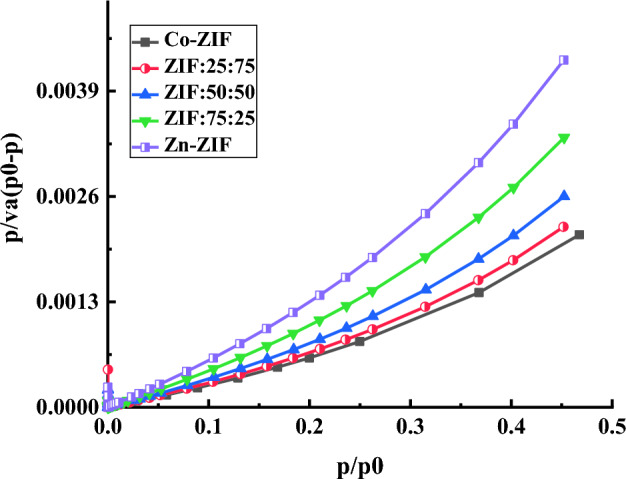
The BET plots of mono- and bi-metallic ZIF samples with zinc or/and cobalt ions with the different molar ratios of Zn to Co salts.

To evaluate the SSA of mono- and bi-metallic ZIF particles with zinc or/and cobalt ions synthesized using different molar ratios of Zn to Co salts, the BET plots represented in Fig. 9 across with the following equation^32,33^ are used and the SSA values of mono- and bi-metallic Zn/Co ZIFs particles are reported in Table 4:

7 $$SSA = \frac{{V_{m} Na}}{m\; \times \;22400}$$

where *N*_*a*_ is Avogadro number (mol^−1^), *a* is the cross-sectional area (nm^2^) of N_2_ gas, *m* is the mass of samples (g), and *V*_*m*_ is proportional inversely to the slope of the linear line fitted to the linear part of BET curves for every mono- and bi-metallic ZIFs in Fig. 9. The SSA of mono- and bi-metallic ZIF samples in Table 4 demonstrates that the high SSA particles were formed in mono- and bi-metallic ZIFs based on Zn or/and Co. Furthermore, the direct dependence of SSA on increasing the Co ions in the Zn-ZIF sample can be observed.

### Thermal stability

**Figure 10:**
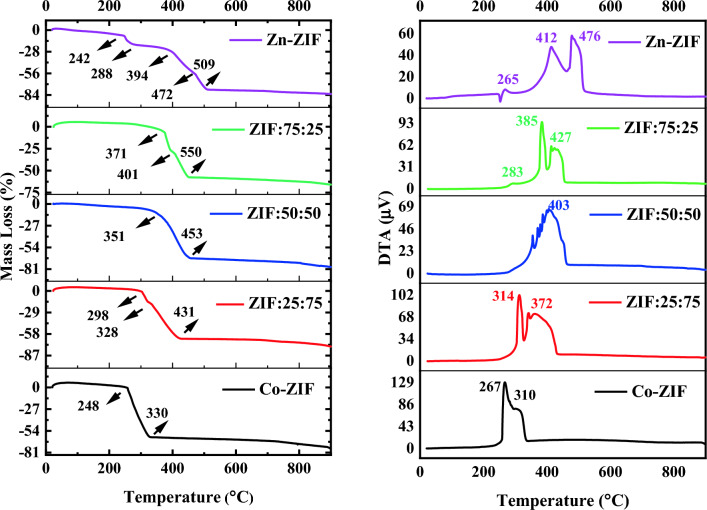
(**a**) thermogravimetric analysis (TGA), and (**b**) differential thermal analysis (DTA) plots obtained for mono- and bi-metallic ZIF particles containing zinc or/and cobalt ions with the different molar ratios of Zn to Co salts.

The thermal stability of materials, determining the temperature of the phase transition occurring in materials, and degradation are required for several applications and should be investigated. Therefore, the results of thermogravimetric analysis (TGA) and differential thermal analysis (DTA) of mono- and bi-metallic ZIF particles synthesized with the different molar ratios of Zn to Co salts are plotted in Fig. 10a,b, respectively. By paying attention to the first part of TGA plots of the mono- and bi-metallic Zn/Co ZIFs, small weight losses can be observed associated with the removal of methanol molecules. Also, it can be concluded that inserting the Co ions into the structures of Zn-ZIF particles and providing bi-metallic Zn/Co ZIF leads to a significant increase of the mentioned region in the TGA curve which means the thermal stability of the synthesized bimetallic Zn/Co ZIF using ammonium is improved significantly. Furthermore, the most thermal stability can be obtained in the ZIF:75:25 sample with a molar ratio of 75% to 25% (~ 55% improvement compared to Zn-ZIF) followed by ZIF:50:50 with the same molar ratio of Zn to Co salts. The mass loss percentage of the Zn-ZIF sample in the mentioned almost constant part of the curve is 8% which changes with increasing the percent of Co ions (6.4%, 9.4%, 2.5%, and 0.5% for the samples ZIF:75:25, ZIF:50:50, ZIF:25:75, and Co-ZIF, respectively). Moreover, different mass loss regions which appear in all synthesized ZIF samples, are related to the endothermic process according to endothermic DTA peaks of the samples as denoted in Fig. 10b. The DTA peaks below 350 °C and their corresponding mass loss in Zn-ZIF and bi-metallic ZIF samples belong to removing some unreacted species from the surface of the ZIF particles. However, the temperature of this DTA peak decreases with increasing the molar ratio of Co salts to Zn salts, and finally, the peak appears at a low temperature (267 °C) for the Co-ZIF sample. This reason was also explained for the mass loss of ZIF-8 in this range of 150–350 °C in reference^34^. The mass loss started from temperatures more than 350 °C for Zn-ZIF and bi-metallic ZIFs (more than 300 °C for Co-ZIF) can be related to the thermal decomposition of 2-MIM and formation of zinc oxide in Zn-ZIF samples, cobalt oxide in Co-ZIF, and the composite of zinc oxide and cobalt oxide in the bimetallic Zn/Co ZIF particles. for Zn-ZIF particles to Co-ZIF particles, the mass losses of the samples in this step are equal to − 77%, − 53%, − 67%, − 64%, and − 61% which indicate decreasing the total mass loss of Zn-ZIF particles in the region by adding the Co ions into its structure and providing bimetallic Zn/Co ZIF particles.

### Water treatment by adsorption and photodegradation

The abilities of synthesized mono- and bi-metallic ZIFs based on Zn or/and Co for water treatment are studied by the adsorption of copper ions as dangerous metal ions. Also, their abilities to photodegrade methylene blue from aqueous solution are investigated under visible light.

#### Dye removal and photodegradation

**Figure 11:**
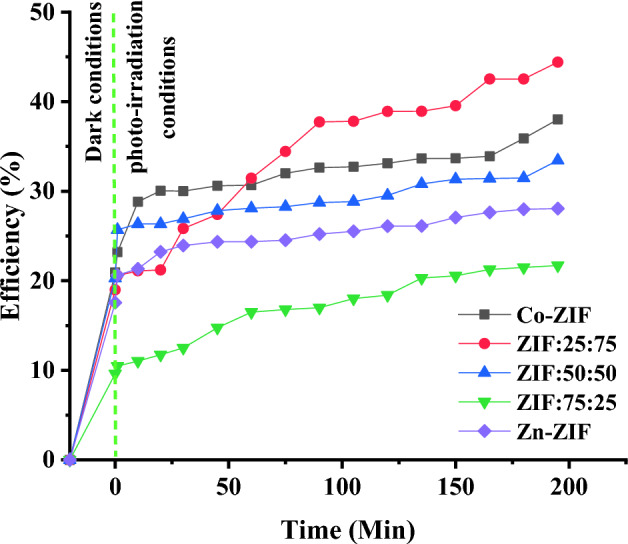
The time-dependent efficiencies of mono- and bi-metallic ZIF particles (1 mg) containing zinc or/and cobalt ions synthesized with the different molar ratios of Zn and Co salts in water treatment from methylene blue (10 ppm) under visible light.

The abilities of synthesized mono- and bi-metallic Zn/Co ZIF samples for water treatment from dye, as nowadays the majority, are investigated. Methylene blue is selected as a toxic, carcinogenic, and non-biodegradable dye that can threaten human health and environmental safety due to its release into natural water sources. Adsorption and photodegradation are effective phenomena in the water purification process. The interaction of light with ZIF particles in a dye solution creates reactive oxygen species (ROS) and provides photodegradation. The efficiency of water treatment from methylene blue under visible light can be calculated by exploring the absorption peak of methylene blue in desirable treatment times using ${\text{Efficiency}}\;{\text{(\% )}}\;{ = }\;{\text{(C}}_{0} - C_{t} )/C_{0} \times 100$^35^, where $C_{0}$ and $C_{t}$ are the initial and time-dependent concentrations of methylene blue in the solution, respectively. The time-dependent efficiencies of mono- and bi-metallic ZIF particles in water treatment under visible light are presented in Fig. 11. It can be figured out from the results that all mono- and bi-metallic ZIF particles can partially separate methylene blue from water in the first 20 min under dark conditions. The water treatment process continues until the efficiencies of mono- and bi-metallic ZIF particles in water treatment are saturated and reach constant values in all samples except for the ZIF:25:75 sample. Also, although the ZIF:25:75 sample has a low methylene blue removal efficiency in dark conditions (~ 17%), it can remove methylene blue by photodegradation in the irradiation condition, and the water purification efficiency reached ~ 45% in 200 min with only a low value of this sample (1 mg). This result can be understood by increasing the SSA, absorption of the sample in the visible region, the pore size distributions, and also increasing the active sites in this sample by adding cobalt to the Zn-ZIF structure which leads to higher photocatalytic activities of the bimetallic Zn/Co ZIF sample.

The important point of the current study can be more obvious if the mass of employed ZIF is carefully considered. The mass of the ZIF particles used in water treatment of 50 cc methylene blue (10 ppm) is very small (only 1 mg) and the efficiency of water treatment reaches up to 45% using ZIF:25:75 which is admirable.

#### Copper ions removal

**Figure 12:**
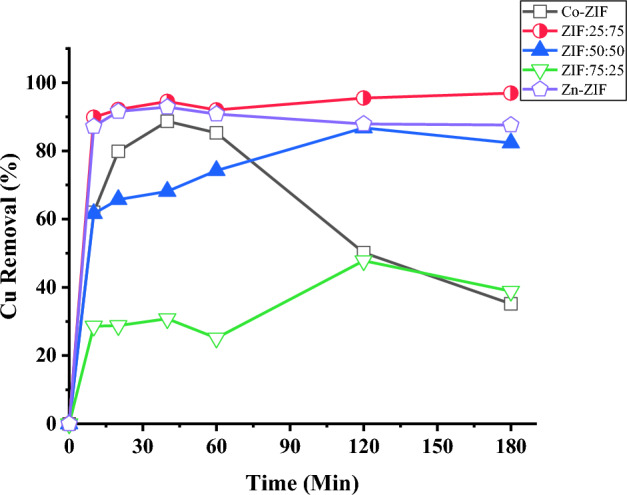
The time-dependent efficiencies of mono- and bi-metallic ZIF samples (1 mg) containing zinc or/and cobalt ions synthesized with the different molar ratios of Zn to Co salts, and used in water treatment from copper ions (10 ppm).

Multifunctional nanoparticles are requested for the simultaneous water treatment from organic dyes as well as metal ions, so in the following, the abilities of the mono- and bimetallic Zn/Co ZIF particles for removal of Cu^2+^ are studied by inserting the ZIF samples in Cu^2+^ solutions (10 ppm) and the efficiency of ZIF particles in water treatment are plotted versus contact time in Fig. 12. As can be observed, high Cu^2+^ removals are obtained by the ZIF particles in low contact time which is related to the high total volumes of micropores formed in the ZIF samples and it is consistent with the hysteresis plots of the ZIF particles in Fig. 7. Also, the results declare that the Zn-ZIF sample and ZIF:25:75 have high copper ion removal (~ 90%) in a very short time of 10 min which is an admirable achievement. Furthermore, the highest Cu^2+^ removal was obtained by the ZIF:25:75 sample (97%) followed by the Zn-ZIF sample (87%) by applying 1 mg/L of the mentioned ZIFs that it is an excellent achievement for the high SSA ZIF particles and shows an interesting improvement obtained by providing bimetallic Zn/Co ZIF. Furthermore, it is worth mentioning that the Cu^2+^ removal of Co-ZIF particles with the highest SSA decreases after 40 min while the same trend is not observed for other samples which can be due to their pore size distribution.

Different efficiencies of ZIF particles in water treatment might be due to factors like SSA of nanoparticles, active sites, zeta potential of the particles, stability and hydrodynamic sizes of the bimetallic ZIFs, pH of polluted water, etc. Therefore, adding the cobalt ions to the structure of Zn-ZIF causes an increase in the SSA of the particles. However, it affects the zeta potential and other effective parameters on the copper ion adsorption leading to a 10 percent improvement in water treatment efficiency of the ZIF:25:75 sample compared to Zn-ZIF and it reaches an admirable value of 97% (almost complete water tratment from copper ions). However, the deep understanding of comparing these samples regarding their adsorption performance needs more investigation.

**Figure 13:**
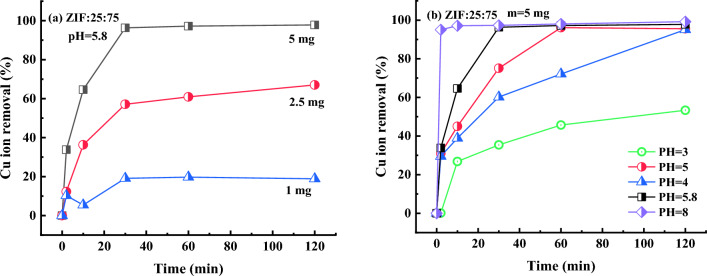
The time-dependent efficiencies of (**a**) different mass of ZIF:25:75 in acidic solutions (pH = 5.8) and (**b**) different solution pH (mass of 5 mg) employed for treatment of highly polluted waters by copper ions (184 ppm).

As mentioned in Section “Dye removal and photodegradation”, the ZIF:25:75 sample shows the highest percentage of water treatment from methylene blue (10 ppm solution) and has the highest Cu^2+^ removal (10 ppm solution). Therefore, it can be concluded the ZIF:25:75 sample can be used for simultaneous treatment of water from both dye molecules (MB) and metal ions (Cu^2+^). In the following, the ZIF:25:75 sample is applied to contaminated water with a high concentration of copper ions (184 ppm) to investigate its efficiency for acidic and highly polluted waters with copper ions. The time-dependent efficiencies of ZIF:25:75 samples with different masses in highly polluted acidic waters with copper ions (184 ppm) are depicted in Fig. 13a which shows increasing the efficiency of water treatment by increasing the adsorbent mass and also the valuable ability of ZIF:25:75 sample in the treatment of high polluted acidic waters with copper ions as a global problem. The treatment efficiency of ZIF:25:75 samples with a mass of 5 mg in acidic water reaches ~ 100% in 30 min making it a suitable material for high polluted water treatment even in acidic conditions.

Due to the excellent performance of the ZIF:25:75 sample in removing copper ions from heavily contaminated water with copper in pH = 5.8, the effect of pH on the water treatment efficiency of the sample is investigated and the results are presented in Fig. 13b. The results declare that the water treatment efficiency of ZIF:25:75 in copper ion removal from water decreases with the decrease in pH of the solution. However, the copper removal efficiencies of these bi-metallic ZIF particles in acidic solution are still excellent and reach ~ 55% in highly acidic conditions (pH = 3), which is better than other reported studies^36^. The comparison of water treatment from MB or copper ions using nano- or micro-structures reported in the scientific literature at different pH values is presented in Table 5 along with the results of the current study which indicates this bi-metallic ZIF:25:75 sample is a suitable candidate for the simultaneous treatment of polluted water from dyes and also low and high concentrations of copper ions.

**Table 5:** The comparison of water treatment from (a) MB or (b) copper ions using nano- or micro-structures reported in the scientific literature at different pH values with (c) the current study.

Sample	Catalyst mass in 50cc (mg)	Dye concentration (ppm)	Degradation (%), treatment time (h)	pH value	Source	References
(a) Methylene blue dye						
AgVO3@ZIF(Zn, Co)	100	20	98.2, 3h	5	Xenon lamp	^37^
Ag-ZnO	50	10	92, 3 h	Neutral	Sunlight	^38^
ZIF-8@N-CQDs/ZIF-67	50	32	65–94, 3 h	4–11	Metal halide lamp	^39^
RGO-Ag-ZnO	50	10	99, 3 h	8.4	Metal halide lamp	^40^
ZIF-67	50	10	80, 3 h	Neutral	Sunlight	^41^
Ga:ZnO	50	10	95, 10 min	Neutral	UVA light	^10^
ZIF-8/NiFe_2_O_4_	50	10	~ 65–94, 3 h	3–9	Visible	^42^
ZnO	10	10	44–83, 3 h	4.3–5.6	UV	^35^

Sample	Catalyst mass in 50cc (mg)	Dye concentration (ppm)	Degradation (%), treatment time (h)	pH value	References	
(b) Copper ions						
ZIF-8	50	100	378.5 mg/g–30 min	5	^43^	
ZIF67@wood	20	100	83.43 mg/L–20 min	6	^44^	
CS-ZIF-8	20–200	100	29-2-98.9, 5 h	5	^45^	
ZIF-8	5	10	94.7, 2 h	Neutral	^46^	
γ-Al_2_O_3_	50	184	98.4, 3 h	5.8	^36^	

Sample	Catalyst mass in 50cc (mg)	Dye concentration (ppm)	Degradation (%), treatment time (h)	pH value	Source light	References
(c) Methylene blue dye and copper ions						
ZIF:25:75 sample (multifunction particle)	1	10 (MB)	~ 45, 3 h	Neutral	Visible	Current work
	1	10 (Cu^2+^)	97, 3 h	Neutral	–	
	5	184 (Cu^2+^)	55–99.3, 2 h	3–8, 2 h	–	

## Conclusion

Water treatment from organic dyes like methylene blue and metal ions like copper ions is important due to their harmful effects on the environment and human health, their existence in drinking water sources or being discharged into the life medium. Methylene blue is a common dye used in various industries, and its release into water bodies can lead to contamination and harm aquatic life. Similarly, metal ions like copper can be toxic to aquatic organisms and may accumulate in the food chain, posing risks to human health. Various physical, chemical, and biological treatment methods are being researched and developed to effectively remove dyes and metal ions from water, making it safe for consumption and reducing environmental impact. Also, different materials were reported to achieve high-efficiency water treatment from the mentioned pollutant to ensure water quality standards are met and to protect ecosystems. MOFs such as zeolitic imidazolate frameworks based on zinc or cobalt have shown great potential for water treatment applications, including the removal of dyes and metal ions like copper ions due to their porous structure and high surface area, which provides a large active surface area for adsorption of dyes and metal ions. However, synthesized ZIF particles based on zinc (Zn-ZIF) have high reflection (HR) in the wide range from UV to NIR regions required for applications needing high reflectance. The results of increasing cobalt to Zn-ZIF declared that the physical properties of nanoparticles specifically optical properties are affected by adding cobalt to the mentioned structures. The highest efficiencies and remarkable multifunctional application (including photocatalytic applications and copper removal) were achieved with bimetallic ZIF particles having a molar ratio of zinc to cobalt salts 25% to 75%, which can candidate them as a powerful and suitable multifunctional material for treating contaminated water by dyes and metal (Cu) ions.

## Data Availability

All data presented in this paper are available upon request by contact with the corresponding author.
